# P50 Systemic lupus erythematous presenting as Kikuchi-Fujimoto disease complicated with haemophagocytic lymphohistiocytosis: a case report and review of the literature

**DOI:** 10.1093/rap/rkae117.081

**Published:** 2024-11-05

**Authors:** Kian Wah Lim, Andreia Ferreira, Emma Davies, Elizabeth Reilly, Shalini Janagan, Matthew Roy, Bashaar Boyce, Robert Marshall, Elizabeth Perry, Anne Sage, Athimalaipet Ramanan, Joanna C Robson

**Affiliations:** Department of Rheumatology, Bristol Royal Infirmary, University Hospitals Bristol and Weston NHS Foundation Trust, Bristol, United Kingdom; Department of Rheumatology, Bristol Royal Infirmary, University Hospitals Bristol and Weston NHS Foundation Trust, Bristol, United Kingdom; Department of Rheumatology, Bristol Royal Infirmary, University Hospitals Bristol and Weston NHS Foundation Trust, Bristol, United Kingdom; Department of Rheumatology, Bristol Royal Infirmary, University Hospitals Bristol and Weston NHS Foundation Trust, Bristol, United Kingdom; Department of Rheumatology, Bristol Royal Infirmary, University Hospitals Bristol and Weston NHS Foundation Trust, Bristol, United Kingdom; Department of Rheumatology, Bristol Royal Infirmary, University Hospitals Bristol and Weston NHS Foundation Trust, Bristol, United Kingdom; Department of Rheumatology, Bristol Royal Infirmary, University Hospitals Bristol and Weston NHS Foundation Trust, Bristol, United Kingdom; Department of Rheumatology, Bristol Royal Infirmary, University Hospitals Bristol and Weston NHS Foundation Trust, Bristol, United Kingdom; Department of Rheumatology, Bristol Royal Infirmary, University Hospitals Bristol and Weston NHS Foundation Trust, Bristol, United Kingdom; Department of Rheumatology, Bristol Royal Hospital for Children, Bristol, United Kingdom; Department of Rheumatology, Bristol Royal Hospital for Children, Bristol, United Kingdom; University of Bristol, Bristol, United Kingdom; Department of Rheumatology, Bristol Royal Infirmary, University Hospitals Bristol and Weston NHS Foundation Trust, Bristol, United Kingdom; College of Health, Science and Society, University of the West of England, Bristol, United Kingdom

## Abstract

**Introduction:**

Kikuchi-Fujimoto disease (KFD) or histiocytic necrotising lymphadenitis is a rare, self-limiting inflammatory condition that can be associated with autoimmune connective tissue disorders (CTD) especially systemic lupus erythematosus (SLE). The aetiopathogenesis remains unclear but it is thought to be triggered by an infection in individuals with autoimmune predisposition leading to T-cells mediated apoptosis and inflammatory cascade. This condition most commonly affects children and young adults of Asian descent with a slight female preponderance although it can affect any age and ethnic groups. Cervical lymphadenopathy and fever are the salient manifestations, and it can be fatal if complicated with haemophagocytic lymphohistiocytosis (HLH).

**Case description:**

A 16 year old girl presented with a 2-month history of persistent fever, progressive bilateral cervical lymphadenopathy, generalised maculopapular rash, angioedema-like facial swelling, oral ulcers and B symptoms. Laboratory investigations revealed pancytopaenia, profound acute phase response and abnormalities consistent with HLH with an HScore of 200 points. A thorough infectious workup was unrevealing apart from serological evidence of past Epstein-Barr virus (EBV) infection. ANA, anti-Sm, anti-RNP and anti-B2GP1 IgG were positive. An ultrasound of her neck showed bilateral multilevel cervical lymphadenopathy and the excision biopsy of her lymph node confirmed features of histiocytic necrotising lymphadenitis and ruled out lymphoma. Notably, there were scattered EBV-positive blasts and the special staining for mycobacteria was negative. A CT scan has also demonstrated bilateral axillary and abdominal lymphadenopathy with no hepatosplenomegaly. There was thrombosis in her right internal jugular vein with no intracranial or cardiac extension of the thrombus.

The patient was admitted to ITU for close monitoring due to concerns about possible airway compromise but she did not require any respiratory support. She received intravenous (IV) methylprednisolone pulses for three consecutive days, mycophenolate mofetil, hydroxychloroquine and treatment dose of enoxaparin with good response and did not require IV immunoglobulins (IVIG) and/or interleukin-1 receptor antagonists. However, she subsequently developed an abscess in her neck which was complicated by Staphylococcus aureus bacteraemia and underwent an emergency incision and drainage of the abscess. She recovered well with IV antibiotics and was followed up in the CTD clinic with continuous psychological support from the rheumatology clinical nurse specialists.

**Discussion:**

KFD most commonly presents with acute to subacute tender cervical lymphadenopathy and fevers which can be associated with other systemic features such as arthralgia, various cutaneous manifestations with great histologic heterogeneity, and neurological manifestations including aseptic meningitis, meningoencephalitis, acute cerebellar syndrome and optic neuritis. Generalised lymphadenopathy has been reported in 22% of cases but hepato- and/or splenomegaly is uncommon (3-5% of cases). Various infectious agents including the EBV have been implicated as the potential trigger of this condition but the evidence remains inconclusive. Our patient’s lymph node biopsy has demonstrated EBV-positive blasts with serological evidence of past EBV infection but a causal relationship between these findings and KFD cannot be established with certainty. It remains a diagnostic challenge to differentiate KFD from the other more sinister pathologies such as lymphoma, various infectious lymphadenitis and autoimmune conditions such as sarcoidosis and Kawasaki disease. Therefore, early excisional biopsy of the affected lymph nodes is essential for establishing the correct diagnosis and may obviate the need for inappropriate treatments.

Both KFD and SLE share a complex clinicopathological relationship as SLE may precede, occur concurrently as in this case or develop later following the diagnosis of KFD. Moreover, lupus lymphadenitis can mimic KFD clinically and these two entities can only be distinguished histologically by the demonstration of haematoxylin bodies in lupus lymphadenitis. Our patient developed thrombosis in her right internal jugular vein which was thought to be secondary to the compressive effect from her cervical lymphadenopathy rather than due the antiphospholipid syndrome as her repeat antiphospholipid screen was negative.

**Key learning points:**

• Clinicians should be aware of this rare clinical entity to make an early diagnosis and avoid unnecessary investigations. Differentiating KFD from the other sinister conditions such as lymphoma remains a diagnostic challenge therefore an early excisional biopsy of the affected lymph nodes would avoid delayed diagnosis and obviate the need for potentially risky treatments. Patients with KFD should be carefully screened and monitored for SLE as it can precede, occur in conjunction with or follow the diagnosis of SLE. Most cases of KFD resolve within 1-6 months although recurrence has been reported in 3-4% and the mortality rate is estimated to be 2.1%. It is crucial to follow up these patients given the increased risk of recurrence which can continue for many years. Despite having a good response to treatments, our patient suffered from significant alopecia and post-inflammatory hyperpigmentation in her skin which has had a great impact on her self-esteem and mental well-being. We would like to highlight in this case the biopsychosocial ramifications of this condition and demonstrate the importance of a multidisciplinary approach in the management of young people with CTD.

